# Eosinophilic dermatosis of hematologic malignancy in patients with chronic lymphocytic leukemia/non-Hodgkin’s B lymphoma: a single center prospective clinico-pathological study

**DOI:** 10.3389/fmed.2023.1231003

**Published:** 2023-08-08

**Authors:** Andrea Michelerio, Carlo Tomasini, Giacomo Fiandrino, Mara De Amici, Marzia Varettoni, Irene Defrancesco, Chiara Cavalloni, Valeria Brazzelli, Federica Derlino, Marco Paulli, Luca Arcaini, Camilla Vassallo

**Affiliations:** ^1^Department of Clinical-Surgical, Diagnostic and Pediatric Sciences, University of Pavia, Pavia, Italy; ^2^Dermatology Clinic, Fondazione IRCCS Policlinico San Matteo, Pavia, Italy; ^3^Department of Molecular Medicine, University of Pavia, Pavia, Italy; ^4^Department of Pathology, Fondazione IRCCS Policlinico San Matteo, Pavia, Italy; ^5^Laboratory of Immuno-Allergology of Clinical Chemistry and Pediatric Clinic, Fondazione IRCCS Policlinico San Matteo, Pavia, Italy; ^6^Division of Hematology, Fondazione IRCCS Policlinico San Matteo, Pavia, Italy

**Keywords:** eosinophilic dermatosis, insect bites, B cell chronic lymphocytic leukemia, non-Hodgkin lymphoma, eosinophils

## Abstract

**Background:**

The pathogenesis of eosinophilic dermatosis of hematologic malignancy (EDHM) is poorly understood. Previously thought to be a hypersensitivity reaction to insect bites, immune dysregulation and cytokine imbalance are now thought to be responsible. Its prognostic significance is unclear.

**Objective:**

To describe the clinical, pathological and immunological findings in a series of oncohematological patients with EDHM.

**Methods:**

An observational prospective cohort study of oncohematological patients receiving a diagnosis of EDHM between April 2017 and December 2018.

**Results:**

A total of 15 patients with EDHM (10 females and 5 males) were identified among 422 oncohematological patients. Disease presentation varied from firm erythematous papules to more polymorphic presentations. The lesions were most prevalent on the exposed sites, 8/15 patients recalled an insect bite. Lesion seasonality was reported in 13/15 patients. IgE levels were elevated in six patients, circulating IL-4 and IL-5 were within a normal range. Twelve out of 15 patients developed skin manifestations after chemotherapy. The infiltrate could be eosinophil-rich or lymphocytic-rich. Interestingly, the histopathologic findings were in accordance with arthropod bites.

**Conclusion:**

A role for insect bites in EDHM is supported by our findings. EDHM may be related to aggressive hematologic disease.

## 1. Introduction

Eosinophilic dermatosis of hematologic malignancy (EDHM) is a chronic, relapsing pruritic skin disorder occurring in patients with various hematologic neoplasms. Although most often associated with chronic lymphocytic leukemia (CLL), EDHM has also been described in acute monocytic leukemia (1), acute lymphoblastic leukemia (1), myelofibrosis (1), chronic myelogenous leukemia (2), large cell lymphoma, mantle cell lymphoma (1, 3–6), MALT lymphoma (5), diffuse large B-cell lymphoma (5), follicular lymphoma (5), small lymphocytic lymphoma (5), lymphoplasmacytic lymphoma (5), marginal zone lymphoma (7, 8), aggressive T-cell lymphoma (9), multiple myeloma/monoclonal gammopathy of undermined significance (9).

A wide variety of clinical presentations has been described, from erythematous papules or nodules (1, 3, 5, 10–14) to blisters and vesicles (4, 11–13, 15–19), urticarial (18–20) or cellulitis like-plaques (21), or cutaneous ulcerations (16).

This dermatosis was initially interpreted as a delayed hypersensitivity reaction to insect bites (10, 12, 17, 20, 22, 23). Many authors, however, have argued against this association in subsequent reports, since most patients failed to recall insect bites (1, 5, 13, 14, 24–27).

The pathogenesis remains poorly understood. A causal role has been attributed to the immune dysregulation accompanying hematoproliferative disorders and a cytokine imbalance (1, 13, 28), specifically an excess of interleukin 4 (IL-4), which plays a major role in type 2 immune responses (29), and of interleukin 5 (IL-5), which facilitates the production, differentiation and migration of eosinophils (29). In East Asian countries a specific form of hypersensitivity to mosquito bites has been described, usually occurring among children and associated with natural killer (NK) cells lymphocytosis and elevated levels of EBV DNA in the peripheral blood (30, 31).

The aim of this observational prospective cohort study is to describe the clinical, pathologic, and immunologic findings of a series of oncohematological patients with EDHM.

## 2. Materials and methods

Patients under follow-up for hematological lymphoid B-cell malignancies at the Hematology Clinic of our hospital between April 2017 and December 2018 were eligible. Patients complaining dermatologic lesions were referred to the Dermatology Clinic for a clinical evaluation. When EDHM was suspected, dermatological clinical data, including lesion seasonality and duration, awareness of insect bites and history of allergies were collected. Data related to the type of hematological disease, as well as prognostic factors such as genetics (immunoglobulin heavy chain gene mutational status, CLL FISH Panel, TP53), therapy and outcome were also collected.

Depending on their number, the skin lesions were classified as rare (≤2), intermediate (3–20), and numerous ≥ 21. The clinical picture was classified as monomorphic or polymorphic based on the type and number of primary lesions present at the same time.

An Analog Visual Scale (VAS) was used to measure itch intensity, from 1 (“no itch”) to 10 (“worst imaginable itch”) (32, 33). Blood samples were taken for allergological, immunological and laboratory studies. In addition to total IgE levels, specific IgE levels were also determined for venoms/salivary antigens of insects and allergens commonly found in our latitudes.

The immunological tests included determination of autoantibodies to BP180 and BP230 and indirect immunofluorescence (IFI) with Salt-Split.

Circulating specific IgG and IgM antibodies against Epstein Barr virus (EBV), Cytomegalovirus (CMV) and Varicella Zoster virus (VZV), as well as circulating DNA copy numbers were assessed.

In addition to routine laboratory tests, our study also performed quantitative tests for eosinophil cationic protein, mast cell-derived tryptase, as well as Interleukin-4 (IL-4) and Interleukin-5 (IL-5). Expanding on the procedures for IL-4 and IL-5, serum titers were assessed in the peripheral blood from all patients involved in the study. To detect and quantify IL-4 and IL-5, we employed a commercial enzyme-linked immunosorbent assay kit (Immunoassay, R&D Systems, Minneapolis, MN), adhering closely to the manufacturer’s instructions. The resultant concentrations of IL-4 and IL-5 were expressed as pg./mL.

For each patient, punch biopsies were performed on recent, unaltered lesions for histological and direct immunofluorescence (DIF). Immunophenotypic study was performed with monoclonal antibodies against B and T cells antigens. Histopathological sections were reviewed by a pathologist (GF) and a dermatologist trained in cutaneous pathology (CT). Each case was evaluated for changes in epidermis, dermis, and hypodermis, distribution, composition, and presence of lymphoid elements, and vessel changes. In each sample we identified representative hot spots with the highest density of eosinophils in the dermal inflammatory infiltrate and calculated eosinophil number per high-power field (HPF; 40x objective, 400x total magnification) to determine the peak eosinophil count (highest number of eosinophils per HPF). Those patients with a final diagnosis other than EDHM were excluded from the study. Patients performed periodic evaluations and data collection stopped on 30 September 2022. All patients provided informed written consent.

## 3. Results

A total of 217 patients with CLL and 205 patients with non-Hodgkin lymphoma (NHL) were visited in the Hematology clinic between April 2017 and December 2018. Among these 12 patients with CLL (8 females and 4 males, 5.5%) and 3 patients affected by B-NHL (2 females and 1 male, 1.4%) were diagnosed with EDHM and included in the study for a total of 15 patients. All patients were adults with a mean age ranging from 53 to 87 years (mean age 70-years-old, median 68 years).

### 3.1. Clinical results

**Figure 1:**
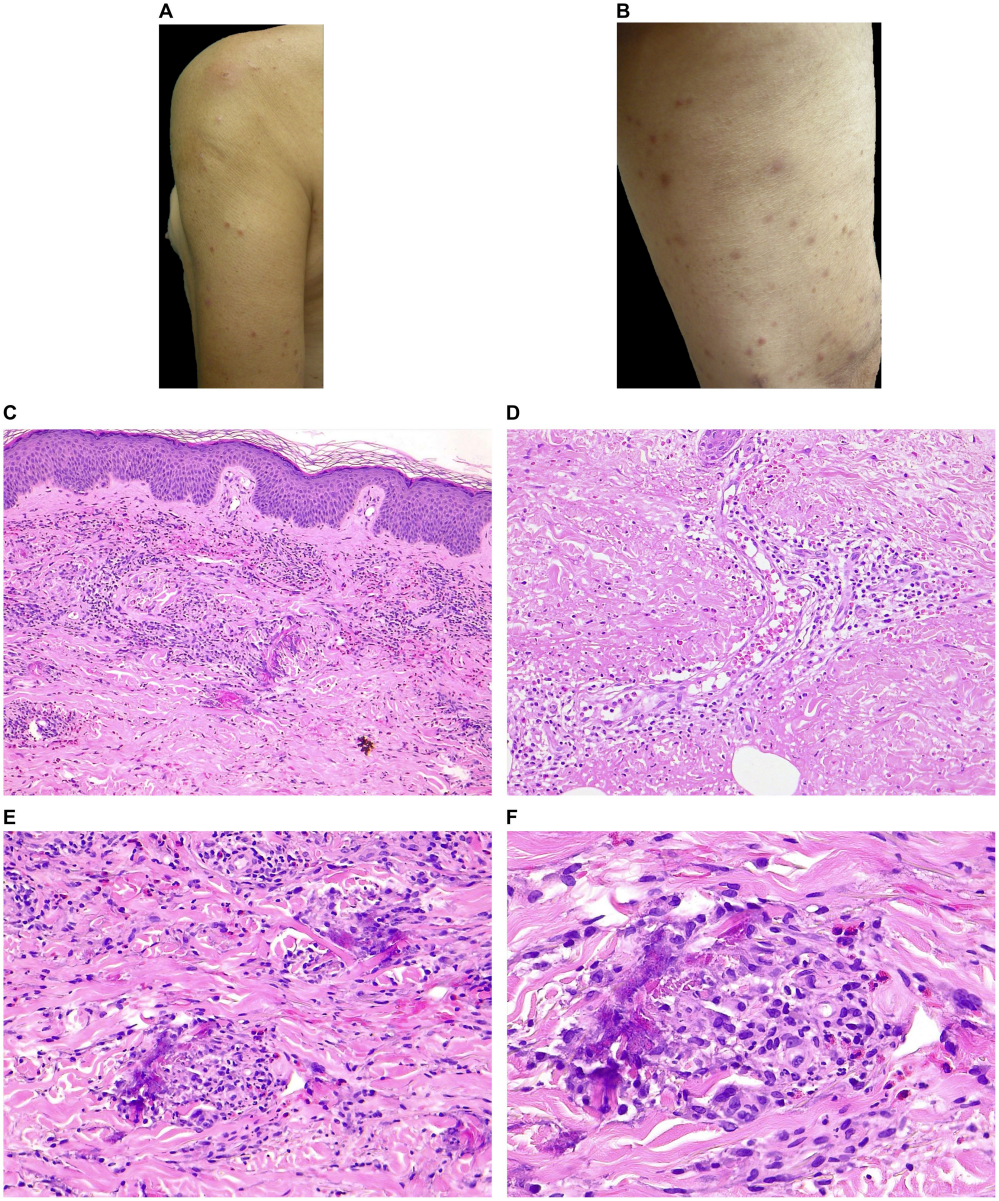
Clinical and histopathological features of EDHM in patient 9. **(A,B)** Monomorphic lesions consisting of erythematous excoriated papules with varying stages of evolution on exposed skin (VAS 7). **(C)** A superficial and deep perivascular, periadnexal, and interstitial infiltrate containing numerous eosinophils in the papillary and mid dermis. **(D)** The sweat glands are involved from the acrosyringium along the sweat ducts to the coiled glands in the deep dermis. Within and around the sweat gland, eosinophils and erythrocytes are visible. **(E,F)** Flame figures with eosinophilic granular deposits are evident in the mid and deep reticular dermis.

**Figure 2:**
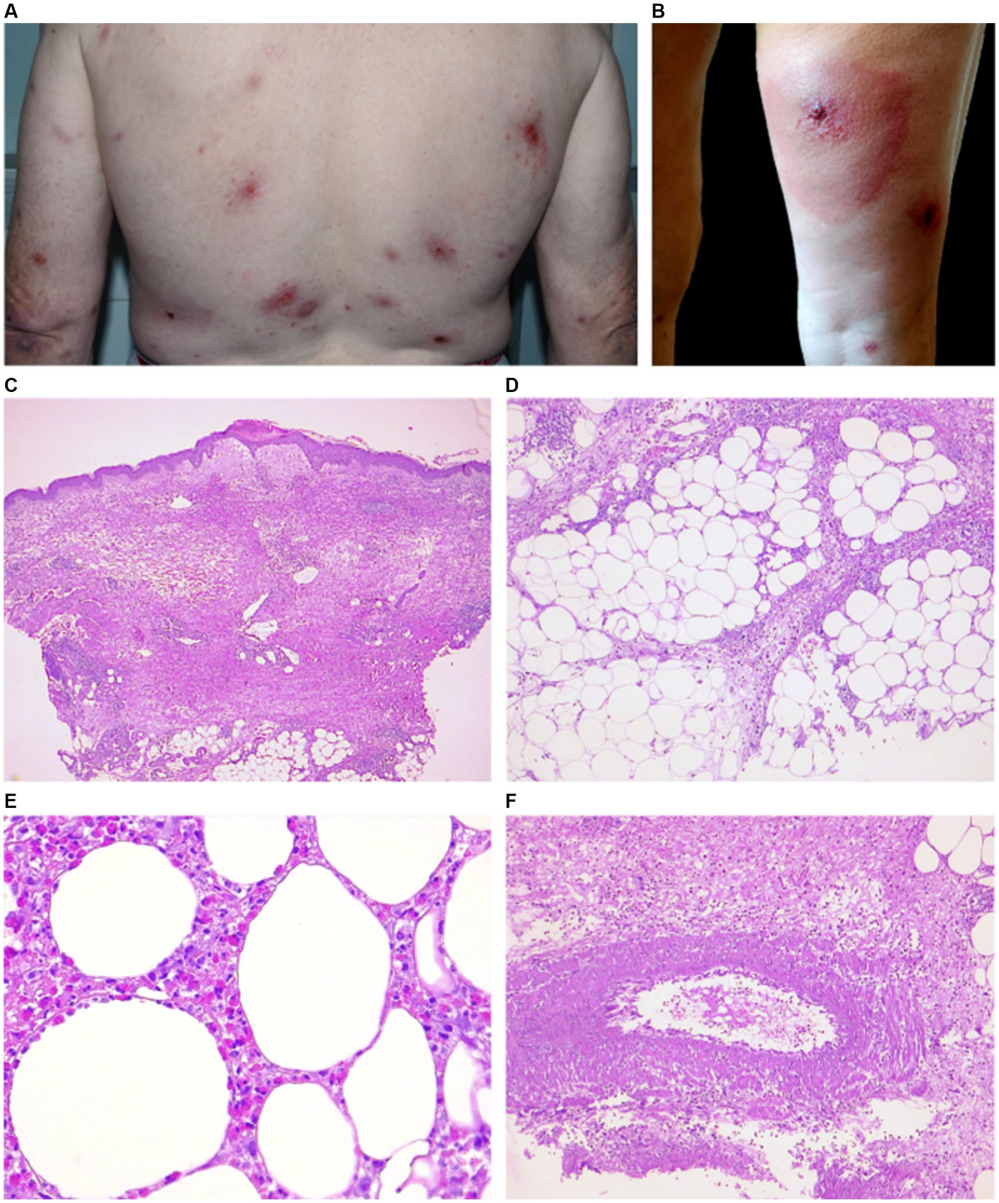
Clinical and histopathological features of EDHM in patient 12. **(A,B)** There are numerous and widespread polymorphic lesions, including erythematous plaques on the trunk and a hypodermitis-like lesion on the right leg (VAS 5). **(C)** A central ulcerated area in the epidermidis, flanked by epidermal spongiosis and a markedly edematous papillary dermis, underlying a superficial and deep dermal eosinophilic inflammatory infiltrate. **(D)** A picture of septal and lobular panniculitis can be seen in the hypodermis due to the infiltrate. **(E)** A high-power view shows a diffuse infiltration of eosinophils in fat lobules. **(F)** An area of leukocytoclastic vasculitis with neutrophils, cytoclasis and focal fibrinoid necrosis in vessel walls can be in the hypodermis.

Clinical and laboratory data of the patients are summarized in Table 1. A monomorphic presentation consisting of firm erythematous papules was observed in 8 patients (53.3%; Figures 1A,B), whereas 7 patients (46.7%) showed polymorphic lesions consisting of erythematous-violaceous or urticarial papules, plaques and nodules and blisters (Figures 2A,B). The lesions were most prevalent on the lower extremities (14/15 cases, 93.3%), followed by the upper extremities (12/15 cases, 80%), the face (6/15 cases, 40%) and the trunk (3/15 cases, 20%).

**Table 1:** Clinical data of the study patients.

Case	Sex	Age (y)	Hematological disease	Stage (Binet if CLL, Ann Harbor if other)	Location	IgHV mutational status	CLL FISH panel	TP53	Main clinical presentation	Lesion number	Itch (VAS scale)	Known bites/seasonality	Allergy	Total IGE	Relation to disease
1	F	77	SLL/CLL	A	Upper and lower extremities, face	Mutated	trisomy 12	wt	Polymorphic; purpuric papules and nodules on the limbs, urticarial plaques on the face	Intermediate	6	Y/Y	*/*	<2	A
2	F	76	CLL	A	Upper and lower extremities, face	Unmutated	del(11q); del(13q)	wt	Monomorphic; urticarial plaques centrally excoriated	Intermediate	9	Y/Y	/	2,49	A
3	F	87	CLL	A	Lower extremities, face	Unmutated	del(13q)	wt	Polymorphic; blisters on the limbs, urticarial plaques on the face	Intermediate	10	N/Y	/	<2	A
4	M	57	CLL	A	Lower extremities	Unmutated	No alterations	wt	Monomorphic; purpuric urticarial plaques centrally excoriated	Rare	8	N/Y	Asthma, seasonal rhinitis	191	A
5	F	76	CLL	A	Upper and lower extremities, face, trunk	Unmutated	No alterations	wt	Polymorphic; purpuric papules and nodules on the limbs, urticarial plaques on the face and trunk	Numerous	10	N/N	/	11,8	A
6	F	77	CLL	A	Upper extremities	Mutated	del(13q)	mut	Monomorphic; urticarial plaques	Rare	7	Y/Y	/	46,4	A
7	F	61	CLL	B	Upper and lower extremities, face	Unmutated	No alterations	mut	Polymorphic; panniculitis-like plaques, excoriated erythematous papules, urticarial plaques	Numerous	10	N/N	Seasonal rhinitis	1,554 H	B
8	F	66	CLL	A	Upper and lower extremities, trunk	Unmutated	Trisomy 12	wt	Monomorphic; centrally excoriated erythematous papules and nodules	Numerous	10	Y/Y	/	310 H	B
9	M	71	FL	III	Upper and lower extremities	NA	NA	NA	Monomorphic; centrally excoriated erythematous papules and nodules	Intermediate	6	Y/Y	Bee sting allergy	13	A
10	F	66	MZL	IV	Upper and lower extremities	NA	NA	NA	Polymorphic; urticarial plaques on the upper extremities, bullous lesions on the lower extremities	Intermediate	6	Y/Y	/	<2	A
11	F	78	FL	IV	Upper and lower extremities	-NA	NA	NA	Polymorphic; erythematous papules, cellulitis-like plaques on the upper extremities	Numerous	6	N/Y	/	966 H	A
12	M	68	CLL	A	Upper and lower extremities, face, trunk	Unmutated	No alterations	wt	Polymorphic; panniculitis-like plaques; urticarial plaques, centrally excoriated erythematous papules	Numerous	5	Y/Y	/	5,000 H	B
13	M	65	CLL	A	Upper and lower extremities	Unmutated	del(13q), del(17p)	mut	Monomorphic; centrally excoriated erythematous papules and nodules	Numerous	5	N/Y	/	24,5	A
14	F	53	CLL	A	Lower extremities	Unmutated	del(13q)	wt	Monomorphic; centrally excoriated urticarial plaques	Rare	6	N/Y	/	496 H	A
15	M	67	SLL/CLL	B	Upper and lower extremities	Unmutated	No alterations	NA	Monomorphic; excoriated vesico-papules	Intermediate	10	Y/Y	Asthma, bee sting allergy	>5,000 H	A

M, male; F, female; SLL, small lymphocyte lymphoma; CLL, chronic lymphocytic leukemia; FL, follicular lymphoma; MZL, marginal zone lymphoma; IgHV, immunoglobulin heavy chain gene; wt, wild type; mut, mutated; NA, not assessed; H, high values; N, no; Y, yes; A, after it was diagnosed; B, before it was diagnosed; Rare, equal or less than 2 lesions; intermediate, more than 2 but less than 21; numerous, equal or more than 21.

Eight of 15 patients (53.3%) recalled the insect bite as the culprit. Lesions were numerous in 6 patients (mean VAS 7,1), intermediate in 6 patients (mean VAS 7,8), and rare in 3 patients (mean VAS 7).

No systemic symptoms were present; 2 patients had slightly elevated erythrocyte sedimentation rate and 4 patients had slightly elevated C-reactive protein.

There was a seasonal variation in the appearance of lesions in 13 patients (86.7%), with eruptions appearing in summer and resolving in autumn. Two cases (#5, #7), which had numerous lesions and a VAS score of 10, continued to exhibit skin manifestations and pruritus until late autumn, and 1 of them case (#7) developed new skin lesions during winter; both these patients experienced relapses only during the summer months.

Four patients (26.7%) had a previous allergy history and IgE levels were elevated in 6 patients (40%). Patient 12 had specific IgE against the Common Mosquito despite a negative allergy history.

None of the patients had detectable VZV DNA, but 13 (86.7%) had anti-VZV IgG antibodies with negative IgM antibodies, one had negative IgG and IgM antibodies (#6), and one had positive IgG and IgM antibodies (#5). In none of the patients was CMV DNA detectable, however all had anti-CMV IgG antibodies with negative IgM antibodies. Only two patients (#12 and #15) had detectable EBV DNA (360 copies/ml and 7,380 copies/ml, respectively). 14/15 patients had anti-EBV IgG antibodies and negative IgM antibodies while only one patient (#5) had positive IgG and IgM antibodies.

In all cases, circulating IL-4, IL-5, complement fractions C3 and C4 were within a normal range. Three patients (#5, #9, #12) had elevated eosinophil cationic protein levels, and 2 (#7 and #14) had borderline levels. In 5/15 patients (#3, #5, #7, #9, #12, 33%), peripheral eosinophilia was present. In 1 case (#5) there was a previous history of eosinophilic facial granuloma.

Among CLL patients, in 3 patients (#7, #8, #12) the cutaneous lesions appeared before the diagnosis of the hematological malignancy (average 14 months), while in 9 cases the dermatosis developed afterwards. With regard to chemotherapy, in 1 case (#14) the cutaneous lesions appeared immediately after the first chemotherapy cycle (fludarabine-cyclophosphamide-rituximab), while in 8 cases appeared several months after the first chemotherapy cycle (range 3–192 months, mean 54 months). At the time of lesion appearance, 3/8 patients were in complete remission and not receiving any treatment for the hematologic malignancy; 2 patients had a relapse, and 3 patients were in partial remission.

All patients with non-Hodgkin B lymphomas had developed dermatologic manifestations after undergoing chemotherapy at an advanced stage of the disease: patients 9 and 11 had follicular lymphoma stage III and IV, respectively, while patient 10 had marginal B lymphoma stage IV. A total of 13 patients received Rituximab treatment and 1 patient received Obinutuzumab treatment.

The average follow-up for the hematological disease was 7 years. The disease progressed or relapsed in 9 patients. Five patients were lost at follow-up while 3 patients died (2 because of infectious complications). A mutational status for the immunoglobulin heavy chain (IgHV) gene was determined in 12 patients, 10 of whom were found to be unmutated. Using the CLL FISH panel, 5 patients were found to have no alterations, 2 patients had trisomy 12, 3 patients had 13q deletions, 1 patient had 13q and 11q deletions and 1 patient had 13q and 17p deletions. Wildtype p53 function was lost in 3 patients, while it was not assessed in 4 patients.

The use of low dosage systemic prednisone (0.5 mg/kg/die), oral antihistamines, and topical high potency steroids resulted in a marked improvement in most cases after 4 weeks. Precautions were advised to patients, including the use of protective clothing and insect repellents and avoiding outdoor activities.

The average duration of dermatological disease was 62 months. During the dermatologic follow-up 8/15 patients experienced relapses during the summer months. An attempt was made to treat patient 8 with omalizumab at a dose of 300 mg every 4 weeks, without success (34).

### 3.2. Histopathology and immunohistochemistry

On histopathological examination, there was epidermal acanthosis (11/15) and spongiosis (11/15) with associated exocytosis of single CD3+ lymphocytes and/or granulocytic elements (14/15).

In the papillary and mid dermis, there was a superficial and deep perivascular (15/15) and periadnexal and interstitial (10/15) infiltrate with eosinophils (Figure 1C). Fourteen out of 15 patients displayed features of periadnexal involvement (either sweat glands or hair follicles). The sweat glands were extensively involved in the inflammatory process, as evidenced by the lymphocytic and eosinophilic infiltration in the acrosyringium, around and within the sweat ducts, as well as in their deep portion of the dermis/subcutis (Figure 1D). The infiltrate involved the hair follicles in 8 cases (perifollicular infiltration). In 12 patients, extravasated erythrocytes were observed.

Eosinophils per HPF ranged from 25 to 200 (mean 90, median 80). No correlation was found between the number of lesions, itching, or IgE level and the number of eosinophils/HPF. Flame figures (5/15), focal necrobiosis (7/15), and eosinophil-associated microgranulomas (3/15) were observed (Figures 1E,F). Interstitial edema was common (10/15) and dermal sclerosis with hyaline collagen degeneration was observed in all cases.

A wedge-shaped infiltrate was observed in 3 cases (Figure 2C) (14, 22). The inflammatory reaction in 11/15 involved also the hypodermis, with lobular and septal eosinophilic panniculitis (Figure 2D). Eosinophilic rimming of subcutaneous fat lobules was observed in 2 cases (Figure 2E). In 8/15 cases vasculitis of medium-size vessels was also observed (Figure 2F).

In 4 cases (#5, #8, #13, #14), the infiltrate was lymphocytic-rich, mostly CD3+, with rare CD30+ and CD20+ lymphocytes. Two cases (#13, #15) showed a small leukemic B-cell component (CD20−/+, CD79a+, CD23+), not exceeding 10% of the infiltrate.

### 3.3. Immunopathologic findings

One patient had low titer serum anti-BP180 antibodies (#12) and one had low titer serum anti-BP230 antibodies (#3). The results of direct immunofluorescence were heterogeneous and non-specific; however, weak perivascular IgM deposits were detected in 13 out of 15 patients (86.7%). There was weak IgG positivity in three out of 15 cases (20%), while weak IgA positivity was found in five out of 15 cases (33.3%). Four patients had perivascular fibrinogen deposits, while eight patients had fibrinogen deposits located in the dermis; two patients had both dermal and perivascular fibrinogen deposits, and one patient had fibrinogen deposits located at the dermo-epidermal junction. In all cases, Salt-Split and indirect immunofluorescence studies were negative.

## 4. Discussion

To date, the pathogenesis of EDHM and its relationship with hematologic malignancies are poorly understood, and the literature consists primarily of case reports and retrospective studies (1, 5, 9, 13, 18, 26). Indeed, the second largest retrospective study in the literature involved 38 patients from a country where the estimated incidence of CLL alone in 2018 was 4,674 (35) and EDHM remains a probably underrecognized condition. In our study, we observed an overall incidence of EDHM of approximately 3.55% in the combined patient population of CLL and NHL.

One of the most controversial issues of EDHM is the role of insect bites. Typically, insect bite reactions present as grouped or disseminated erythematous, pruritic papules on exposed areas, but may develop into a longer lasting popular urticaria or a generalized and pleomorphic eruption (36).

Our results support a key role of insect bite in the pathogenesis of EDHM. In most cases, the lesions were on exposed areas, whereas the trunk was only affected in three (20%) cases, in which atopy (two of the cases had elevated IgE levels) or a particular insect (e.g., bed bugs) could have contributed to a wider distribution of lesions. Furthermore, in the majority of our patients (86.7%) seasonality was documented, while in only two patients did the lesions or itching persist till late fall and early winter. As our geographical area is characterized by hot and humid summers and cold winters, insects (common mosquitos) are expected mostly in summer. The interpretation of data is more difficult in other geographical contexts, such as Israel, which has cool, rainy winters and hot, dry summers, and specific mosquito populations are present throughout the year (1, 26, 37).

In our study more than half of the patients recalled insect bites as the cause of the lesions. This contrasts with most EDHM reports, which showed no history or response to preventive measures supporting insect bites (2, 5, 10, 12, 13, 17, 20, 22, 23). In this regard, it should be noted that the bites are usually painless and the skin reaction can be delayed for days (38). Furthermore, patients may be reluctant to accept insects as responsible for their dermatosis (39) and even Davis reported a patient with linear lesions denying being bitten (13). Moreover, reactivation of old lesions with itch in response to new bites may limit the efficacy of preventive measures (40). This effect is thought to be secondary to circulating insect antigen stimulating cutaneous T cells in previously sensitized sites (40).

A pathogenetic role for insect bites is also supported by the histopathology. The classical histopathologic hallmark of an arthropod bite is a superficial and deep, perivascular, periadnexal, and interstitial inflammatory dermal infiltrate composed of lymphocytes and eosinophils, often in association with an overlying focus of spongiosis, sometimes evolving into a vesicle or even progressing to epidermal necrosis (41, 42). The histopathologic findings of the current study are in accordance with those previously published about arthropod bite (41, 42). Moreover, 14 out of 15 patients displayed features of periadnexal involvement (either sweat glands or hair follicles). Ackerman et al. suggest that insect bite reactions have a periadnexal distribution of infiltrate because, attracted by microbes and lipids in sebaceous glands, insects bite at hair follicle ostia, causing folliculocentric infiltrates (41). The wedge-shaped infiltrate and involvement of the acrosyringium suggest arthropod bite reactions, with the latter due to sweat attractiveness to mosquitoes and other insects (42, 43).

EDHM pathogenesis and prognostic significance were also investigated. In accordance with literature, the majority of patients were diagnosed with CLL. The higher incidence of CLL could be attributed to the dysfunctional T-cell compartment in CLL patients showing a T-helper Th2/Th1 ratio imbalance with an aberrant recruitment of a Th2-dominant response (44, 45). The Th2 response polarization may also occur in the skin as a result of a specific cytokine microenvironment that promotes EDHM formation through eosinophil recruitment. To investigate a possible role of atopy in this Th2 response polarization, we assessed a previous allergy history and the IgE levels of our patients. Six of them had elevated levels of IgE, which may be responsible for driving both eosinophilic granulocytes, as well as certain scattered lymphoid elements of CLL, which are characterized by the expression of CD23, a low-affinity IgE receptor (46). This may be particularly pertinent to Patient 12, who showed high level of total IgE and specific IgE against the Common Mosquito. Patient 12 presented with diffuse and pleomorphic cutaneous manifestations, and histopathologically a dense eosinophilic infiltrate with panniculitis and vasculitis. Our study found no excess of serum levels of IL-4 and IL-5, which play a role, respectively, in type 2 immune responses and eosinophil regulation (29), however, these interleukin levels were not tested in skin lesions, which is a limitation of the study. Few anecdotal reports of the use of monoclonal antibody blocking IL-4 and IL-13 (Dupilumab) in the treatment of EDHM show discordant outcomes that neither confirm nor contradict the possible role of IL-4 (47–50).

A role in EDHM development may also be played by chemotherapy. A total of 12 out of 15 patients in our study developed skin manifestations after chemotherapy and, interestingly, 11 received Rituximab treatment. Rituximab has been shown to reduce serum IgG4 (51), which is known to have anti-inflammatory properties, and whose reduced level might lead to more severe reactions to insect bites (52).

Finally, in East Asia countries hypersensitivity to mosquito bites occurs most commonly in children and has a distinctive pathophysiology. When disease occurs, patients have NK-cell lymphocytosis and high EBV DNA load (typically above 1,000 copies/μg DNA) in the peripheral blood, and may progress to systemic diseases, such as hemophagocytic lymphohistiocytosis, chronic active EBV disease, and EBV-associated malignancies (31). Our findings do not support a role for EBV in the evolution of the dermatologic disease and these observations may reflect different genetic and epigenetic factors of EBV infection between East Asia and Western countries.

Another matter of debate is the prognostic value of EDHM in hematologic malignancies. As previously mentioned, the NHL patients were in advanced stages of the disease when their skin lesions appeared and, in general, all patients in the study required chemotherapy during the course of their hematologic disease, including those developing EDHM before the hematological diagnosis. The fact that CLL generally has an indolent course and is treated in a limited number of cases may indicate that EDHM indicates a more aggressive course of the hematologic disease. Where data are available, a similar trend has been observed in the literature as well (1, 3, 13). It is noteworthy that 10/12 CLL patients had an unmutated IgHV gene, which is associated with worse prognosis (53). Moreover, unmutated IgHV CLL cells generally produce less IL-10, an immunosuppressant molecule, which may explain the exaggerated reactions and the higher incidence of EDHM among CLL patients compared to other hematologic malignancies (54). Finally, the shift to a Th2-dominant response, which has been hypothesized to contribute to EDHM, is correlated with the progression of CLL, and type 2 cytokines facilitate the escape of cancer cells from the immune system (44).

EDHM is named after the eosinophilic infiltrate found in the tissue, and our findings support the hypothesis that EDHM is part of the reaction pattern associated with Wells’ syndrome. Wells’ syndrome (WS) is a rare dermatosis characterized by a variety of itchy cutaneous manifestations that are usually recurrent over several years (43). It can present in morphologically varying forms, such as a plaque-type lesion, annular granuloma-like, urticaria-like, papulovesicular, bullous, papulonodular, or fixed drug eruption-like (43), and different lesions may present simultaneously in a patient (55). It is often located on the extremities (55) and has been associated with various diseases, including hematologic diseases and arthropod bites (56–58). Peripheral eosinophilia, leukocytosis, or elevated inflammatory markers may be observed (57).

Histopathological findings of WS not specific and include subepidermal edema, granuloma, and frequent flame figures. According to the current definition, vasculitis is not found in WS, but vasculitic features have actually been seen in a few patients (58). On a pathophysiologic level, the aforementioned clinical variability might be explained by the varying degree of eosinophil infiltration and degranulation. As a result, infiltration that results in only mild degranulation will produce a plaque-like appearance. Conversely, if a greater amount of mediators and toxic granules are released, urticarial, vesicular, or lesions will develop due to vasodilation and additional tissue damage (59). Our patients also exhibited this clinical polymorphism, even with blisters and urticarial plaques, which could explain some of the “pemphigoid-like eruptions” reported by some Authors in association with CLL (18).

It is interesting to note that one of our patients had a history of granuloma faciale, an eosinophilic dermatosis associated with vasculitis, supporting a role for a nonspecific eosinophilic hypersensitivity reaction to either endogenous or exogenous stimuli in a continuum spectrum.

In conclusion, the results of our study support the role of insect bites as a trigger for EDHM in the context of an adaptive immune response dysfunction, either as a result of the disease itself or as a consequence of chemotherapy. In addition, we hypothesize that EDHM may have a negative prognostic value and in some cases represent a hallmark for disease progression, suggesting the need for closer follow-up, although the size of our sample does not allow for definitive conclusions.

Furthermore, we propose a potential pathogenetic link between EDHM and other eosinophilic dermatoses, particularly Wells syndrome. Further studies are needed to clarify the pathogenetic mechanisms underlying this disorder as well as its prognostic value.

## Data availability statement

The original contributions presented in the study are included in the article/supplementary material, further inquiries can be directed to the corresponding authors.

## Ethics statement

The studies involving human participants were reviewed and approved by Comitato Etico Referente per l’Area di Pavia. The patients/participants provided their written informed consent to participate in this study. Written informed consent was obtained from the individual(s) for the publication of any potentially identifiable images or data included in this article.

## Author contributions

AM, CT, FD, and CV contributed to conception and design of the study. AM organized the database. AM, CT, GF, MA, MV, ID, CC, VB, FD, MP, and LA contributed to the collection of data. AM, FD, and CV performed the data analysis. AM wrote the first draft of the manuscript. All authors contributed to the article and approved the submitted version.

## Funding

Open access funding provided by Ricerca Corrente Ministero della Salute—Fondazione IRCCS Policlinico San Matteo.

## Conflict of interest

The authors declare that the research was conducted in the absence of any commercial or financial relationships that could be construed as a potential conflict of interest.

## Publisher’s note

All claims expressed in this article are solely those of the authors and do not necessarily represent those of their affiliated organizations, or those of the publisher, the editors and the reviewers. Any product that may be evaluated in this article, or claim that may be made by its manufacturer, is not guaranteed or endorsed by the publisher.
